# Dietary saturated fat and fibre and risk of cardiovascular disease and all-cause mortality among type 1 diabetic patients: the EURODIAB Prospective Complications Study

**DOI:** 10.1007/s00125-012-2550-0

**Published:** 2012-04-12

**Authors:** D. A. J. M. Schoenaker, M. Toeller, N. Chaturvedi, J. H. Fuller, S. S. Soedamah-Muthu, the EURODIAB Prospective Complications Study Group

**Affiliations:** 1 Division of Human Nutrition, Wageningen University, P.O. Box 8129, 6700 EV Wageningen, The Netherlands; 2 Department of Endocrinology, Diabetology and Rheumatology, University Hospital, Heinrich-Heine-University, Düsseldorf, Germany; 3 International Centre for Circulatory Health, National Heart and Lung Institute, Imperial College London, London, UK; 4 Department of Epidemiology and Public Health, University College London, London, UK

**Keywords:** All-cause mortality, Cardiovascular disease, Dietary fibre, Nutrition, Replacement of saturated fat, Saturated fat type 1 diabetes

## Abstract

**Aims/hypothesis:**

Low adherence to recommendations for dietary saturated fatty acid (SFA) and fibre intake in patients with type 1 diabetes mellitus may heighten their increased risk of cardiovascular disease (CVD) and mortality. We examined the relationship of SFA and total, soluble and insoluble fibre with incident CVD and all-cause mortality in type 1 diabetic patients.

**Methods:**

A prospective cohort analysis was performed in 2,108 European type 1 diabetic patients aged 15–60 years who were free of CVD at baseline and enrolled in the EURODIAB Prospective Complications Study (51% male). Diet was assessed from a standardised 3 day dietary record. HR were calculated using Cox proportional hazards models.

**Results:**

During a mean follow-up of 7.3 years, 148 incident cases of fatal and non-fatal CVD and 46 all-cause deaths were documented. No statistically significant association was found between SFA and CVD and all-cause mortality. Total dietary fibre, per 5 g/day, was associated with lower all-cause mortality risk (HR 0.72; 95% CI 0.55, 0.95). This association was stronger for soluble fibre (per 5 g/day, HR 0.34; 95% CI 0.14, 0.80) compared with insoluble fibre (per 5 g/day; HR 0.66; 95% CI 0.45, 0.97). Similar results were found for the association with CVD.

**Conclusions/interpretation:**

This study suggests that reported dietary SFA is not significantly associated with CVD and all-cause mortality in type 1 diabetic patients. On the contrary, higher dietary fibre consumption, especially soluble fibre, within the range commonly consumed by type 1 diabetic patients, may contribute to the prevention of CVD and all-cause mortality in type 1 diabetic patients.

**Electronic supplementary material:**

The online version of this article (doi:10.1007/s00125-012-2550-0) contains peer-reviewed but unedited supplementary material, which is available to authorised users.

## Introduction

Patients with type 1 diabetes mellitus are at a markedly increased risk of cardiovascular disease (CVD) [1, 2]. The most important modifiable risk factors for CVD, hypercholesterolaemia and hypertension, are more common in diabetic patients than in the general population [3, 4]. Diet is an important modifiable factor that is related to CVD risk factors [5, 6]. A focus of dietary recommendations for both CVD and diabetes prevention and treatment has been a reduction in saturated fatty acid (SFA) intake and an increase in dietary fibre intake [7, 8]. However, low adherence to dietary recommendations (<10% of energy intake from SFA and 20 g/1,000 kcal for dietary fibre) [9], and even less healthy eating habits compared with a non-diabetic population [10], may heighten the increased risk of CVD in patients with type 1 diabetes [11]. Both nutrients are important, as it has been shown that low SFA intake is often accompanied by high dietary fibre intake and a healthy lifestyle [12], which might affect the association with CVD risk, as suggested from cross-sectional studies [13, 14]. In type 1 diabetic patients an independent association has been shown between dietary fibre intake and serum cholesterol (in men) and CVD (in women) [14].

A recent systematic review [15] and meta-analysis [16] found that there was no statistically significant evidence of an association between SFA intake and CVD risk in the general population, even though several weaknesses in the meta-analysis may question the validity of the results [17, 18]. Besides, neither the systemic review nor the meta-analysis separately evaluated whether intake of SFA may have a different association with CVD risk depending on the nutrient being replaced. Evaluation of the association between SFA intake and CVD, however, requires replacement by other macronutrients to maintain energy balance.

The importance of adequate dietary fibre intake in the prevention and treatment of CVD is well established in the general population. Results from a pooled analysis of cohort studies [19] and a review [20] suggest an inverse association between dietary fibre intake and coronary heart disease risk in the general population. In the Nurses’ Health Study, reduced all-cause and CVD-specific mortality was found in type 2 diabetic patients with higher wholegrain and bran intakes [21]. No intervention trials have explored the effect of dietary fibre intake on CVD or all-cause mortality risk.

Little information on nutritional intake is available from large cohorts of type 1 diabetic patients sufficiently followed up for CVD and deaths. Possible relationships with dietary SFA and fibre have not yet been established in this population. Therefore the aim of the present study was to investigate the relationship between dietary SFA and total, soluble and insoluble fibre and incident CVD and all-cause mortality in patients with type 1 diabetes.

## Methods

### Study design

The EURODIAB Prospective Complications Study (PCS) is a clinic-based prospective cohort study including 3,250 individuals with type 1 diabetes. Participants were recruited from 31 centres in 16 European countries and examined between 1989 and 1991. The prevalence of diabetic complications was measured and risk factors associated with these complications were explored. Each centre selected a random sample of patients stratified by sex, age and diabetes duration. Details on patient selection and the standardised methodology have been published elsewhere [22, 23]. Type 1 diabetes was defined as diabetes diagnosed before the age of 36 years with a continuous need for insulin within 1 year of diagnosis. Of those invited, 85% participated. Pregnant women and those with a diabetes duration of less than 1 year were excluded. Ethics committee approval conforming to the Declaration of Helsinki was obtained at each centre; all participants provided written informed consent.

### Study population

Seven years after the baseline examination, study participants were invited for re-examination. Of the 3,250 participants at baseline, 1,142 were excluded for the following reasons: no participation in follow-up examination (*n* = 464), known CVD at baseline (*n* = 238), no information on SFA and dietary fibre at baseline (*n* = 247), and no information on CVD at follow-up (*n* = 193), leaving 2,108 participants available for analysis.

### Assessment of nutrients

Dietary nutrients were determined at baseline (1989–1991) from a standardised 3 day dietary record [24]. Dietary records comprising 2 workdays and 1 day free of work (Saturday or Sunday) were completed by participants according to instructions from a local dietitian. These records were coded by a dietitian using a centrally prepared EURODIAB food list and finally rechecked and computed at the Nutrition Coordinating Centre in Düsseldorf. Pilot study checks were conducted to establish how well we could approximate the intake of 7 days of the week with a 3 day dietary record. No differences were found in the proportion of nutrients between the days. Total energy was calculated using Atwater factors [25]. The nutrients fat (total fat, SFA, monounsaturated fatty acids [MUFA] and polyunsaturated fatty acids [PUFA]), dietary fibre (total, soluble and insoluble fibre), carbohydrates (CH), protein, alcohol and total energy (including energy derived from alcohol consumption) were calculated using the EURODIAB nutrient database [26]. The program could not provide quantities of *trans*-fatty acids (TFA), as complete information on the consumed foods was not available.

A representative sample of the total EURODIAB cohort was randomly selected to test repeatability of the nutritional data [24]. Patients were asked to fill in a second 3 day dietary record 3 weeks after completing the first 3 day record. Repeatability was measured by comparing mean differences with SD for protein, fat (including SFA), CH, dietary fibre, alcohol and energy from the first and second 3 day dietary record. Mean differences in nutrients from the first and second 3 day dietary record were 0.5 g (SD 12.7; 95% CI −1.2, 2.2) for SFA and −0.2 g (SD 5.5; 95% CI −0.9, 0.5) for dietary fibre. A high degree of repeatability was demonstrated and plausible energy values were recorded by 90% of the patients, thus supporting its validity.

### Assessment of fatal and non-fatal CVD and all-cause mortality

Presence of CVD at follow-up was defined as a positive medical history of a cardiovascular event including myocardial infarction, angina pectoris, coronary artery bypass graft surgery or stroke, and/or by electrocardiograms suggestive of probable or possible ischaemia determined from centrally Minnesota-coded electrocardiograms [27]. All CVD events were captured by questionnaires with additional supporting information from hospital records, death certificates and other healthcare documents. Data on mortality were collected at follow-up from hospital case notes or other sources of clinical data at every participating centre. Information on the cause of death was reported by the physician or extracted from hospital records when death certificates could not be obtained. Causes of death were coded according to the ICD-9 (www.icd9data.com/2007/Volume1/240-279/250-259/250/default.htm).

### Assessment of potential confounders

Triacylglycerol and total and HDL-cholesterol were determined using fasting serum and standard enzymatic methods (Boehringer Mannheim, Lewes, UK). LDL-cholesterol was calculated using the Friedewald equation [28]. HbA_1c_ was measured by enzyme immunoassay using a monoclonal antibody (The Royal London Hospital, London, and Dako, Ely, UK). HbA_1c_ values obtained were converted to Diabetes Control and Complications Trial (DCCT; %) [29] and International Federation of Clinical Chemistry and Laboratory Medicine (IFCC; mmol/mol) values in line with the recommendations of the European Association for the Study of Diabetes (EASD) [30]. Resting blood pressure was measured twice with a random zero sphygmomanometer (Hawksley, Lancing, UK). Information on diabetes duration, daily insulin dose and frequency, physical activity and smoking status was gathered from a general questionnaire on lifestyle, disease history and family history completed by all participants. Alcohol was assessed from the 3 day dietary record. Weight and height were measured with indoor clothing without shoes, and BMI was calculated.

### Statistical analysis

All descriptive data were expressed as mean ± SD or median and interquartile range (IQR). The nutrient residual method by Willett was used to adjust nutrients for total energy [31]. Energy-adjusted nutrients are computed as the residuals from the regression model, with total energy as an independent variable and the absolute nutrient as a dependent variable. The nutrient residuals provide a measure of nutrient intake uncorrelated with total energy intake [31]. For descriptive purposes, the nutrient densities (energy percentage [en%]) were calculated as a ratio of nutrient content (g/day) to total energy content (in kJ).

To examine associations between baseline SFA and dietary fibre (total, soluble and insoluble) and incident fatal and non-fatal CVD and all-cause mortality we used Cox proportional hazards analyses to estimate HR and 95% CI. Survival time for each participant was defined as the time between the date of study entry and the occurrence of a CVD event, death, loss to follow-up, or the end of the study, whichever came first. SFA and dietary fibre were energy-adjusted by the residual method and divided into tertiles, with the lowest category as the reference group, to explore different nutrient values and trends. To retain power, SFA and dietary fibre were also analysed as continuous variables. We used three models to adjust for confounders. In model 1, estimates were adjusted for age, sex and energy (kJ/day). In model 2, diabetes duration (years), HbA_1c_ (%), smoking status (number of non-smokers, previous or current smokers), physical activity (physical inactivity, mild physical activity ≥1 time/week, moderate physical activity ≥1 time/week, vigorous physical activity ≥1 time/week) and alcohol (0, >0–<5, 5–<15, 15–<30, 30–<40 and ≥40 g/day) were added. In model 3, adjustments for total dietary fibre (g/1,000 kcal) for the relationships of SFA with CVD and all-cause mortality, or SFA (en%) for the relationships of dietary fibre with CVD and all-cause mortality, were added. To show the robustness of our results, final adjustments were made for MUFA and PUFA (g/day), antihypertensive use (yes or no), daily insulin dose (units/kg) and frequency (number of injections per day). Finally, potential mediators such as systolic blood pressure, total/HDL-cholesterol ratio and BMI were explored to demonstrate potential pathways.

We carried out replacement models [32] to investigate whether a 5% higher energy intake from MUFA, PUFA or CH should replace a 5% lower energy intake from SFA to prevent CVD. Two models were used. Model 1 included age, sex, MUFA, PUFA, TFA, CH and protein expressed as percentages of total energy, and total energy (kJ/day). TFA was calculated as the difference between total fat and major groups of fatty acids (SFA, MUFA and PUFA), as exact values for TFA were not available. Model 2 included variables in model 1 and confounders similar to those of model 2 as described above. The estimated HR for MUFA or PUFA and CH can be interpreted as the estimated difference in risk of a 5% lower energy intake from SFA and a concomitant higher energy intake from MUFA, PUFA or CH.

Possible effect modification by age was tested and a *p* value for interaction <0.10 was considered statistically significant. Including an interaction term to identify modification by centre was not possible, since we had 31 centres in this study in 16 European countries with unequal numbers of participants per centre or country. We did not have a sufficient number of cases for stratified analyses. Data analysis was performed using SAS software (version 9.1; SAS Institute, Cary, NC, USA). Two-sided *p* values <0.05 were considered statistically significant.

## Results

### Study characteristics

Electronic supplementary material (ESM) Table 1 shows a comparison of CVD risk factors between participants excluded from the present study and participants remaining for analyses. Excluded participants appeared to have higher SFA and lower dietary fibre consumption, but a higher CVD risk profile with increased age, diabetes duration, HbA_1c_, systolic blood pressure, BMI, total/HDL-cholesterol ratio, physical activity and smoking.

During a median follow-up period of 7.4 years (IQR 6.9–7.8 years), 148 fatal and non-fatal CVD incident cases and 46 all-cause deaths occurred among 2,108 participants (51% male). Among men and women at baseline, the median age was 31 years (IQR 25–38), the median diabetes duration was 13 years (IQR 7–19), the median SFA intake was 13.5 en% (IQR 11.4–16.1) and the median total dietary fibre intake was 8.2 g/1,000 kcal (IQR 6.0–10.7). Median intakes were 5.7 g/day (IQR 4.3–7.3) for soluble fibre and 12.4 g/day (IQR 9.4–15.9) for insoluble fibre.

Risk factors for CVD according to baseline SFA are given in ESM Table 2. SFA was positively associated with being female, diabetes duration, smoking, alcohol and moderate physical activity, and negatively associated with dietary fibre.

Risk factors for CVD according to baseline total dietary fibre are given in Table 1. Participants in the highest tertile for dietary fibre were more likely to be female, more physically active, smoked less and had lower values for alcohol compared with participants in the lowest tertile. On the other hand, an increase in use of antihypertensive medication was seen over tertiles of total dietary fibre.

**Table 1:** Baseline characteristics of type 1 diabetic patients in the EURODIAB PCS according to tertiles of dietary fibre intake (*n* = 2,108)

Characteristic	Tertiles of fibre intake^a^			*p* value for trend
	1	2	3	
*n*	702	703	703	
Mean follow-up, years	7.3 ± 0.9	7.3 ± 1.1	7.2 ± 1.0	0.03
Age, years	31.0 ± 9.1	32.3 ± 9.8	32.9 ± 10.1	0.0003
Sex, *n* (%) male	393 (65.0)	330 (46.9)	358 (50.9)	0.06
Diabetes duration, years	13.1 (7.8–18.7)	13.0 (7.3–19.7)	12.8 (6.7–20.3)	0.41
HbA_1c_, % (mmol/mol)	8.3 ± 2.0 (67.2)	8.0 ± 1.8 (63.9)	8.0 ± 1.9 (63.9)	0.003
Daily insulin dose, units/kg	0.7 (0.6–0.8)	0.7 (0.5–0.8)	0.6 (0.5–0.8)	0.002
Daily insulin frequency, injections/day	2.0 (2.0–3.0)	3.0 (2.0–3.0)	3.0 (2.0–3.0)	0.03
Systolic blood pressure, mmHg	119.1 ± 16.4	120.2 ± 16.8	120.8 ± 17.1	0.06
Diastolic blood pressure, mmHg	75.1 ± 11.2	75.0 ± 11.2	75.1 ± 11.2	0.9
Antihypertensive use, *n* (%)	42 (6.0)	54 (7.8)	63 (9.0)	0.03
BMI, kg/m^2^	23.6 ± 2.8	23.4 ± 2.6	23.4 ± 2.9	0.16
Triacylglycerol, mmol/l	0.9 (0.7–1.3)	0.9 (0.7–1.3)	0.8 (0.7–1.1)	0.02
Total cholesterol, mmol/l	5.4 ± 1.1	5.3 ± 1.1	5.3 ± 1.2	0.05
HDL-cholesterol, mmol/l	1.5 ± 0.4	1.5 ± 0.4	1.5 ± 0.4	0.51
LDL-cholesterol, mmol/l	3.4 ± 1.0	3.3 ± 1.0	3.3 ± 1.0	0.15
Total/HDL-cholesterol ratio, mmol/l	3.4 ± 1.4	3.4 ± 1.1	3.4 ± 1.2	0.28
Physical inactivity, *n* (%)	15 (2.2)	18 (2.6)	27 (3.9)	<0.0001
Mild physical activity ≥1 time/week, *n* (%)	230 (33.2)	241 (34.7)	195 (28.2)	<0.0001
Moderate physical activity ≥1 time/week, *n* (%)	237 (34.3)	214 (30.8)	241 (34.8)	0.24
Vigorous physical activity ≥1 time/week, *n* (%)	210 (30.4)	222 (31.9)	229 (33.1)	<0.0001
Ever-smoker, *n* (%)	382 (54.4)	332 (47.2)	304 (43.2)	<0.0001
Alcohol intake, g/day	2.4 (0.0–13.0)	1.0 (0.0–8.8)	0.0 (0.0–5.3)	<0.0001
None, *n* (%)	293 (41.7)	338 (48.1)	392 (55.8)	<0.0001
≤20 g/day, *n* (%)	290 (41.3)	275 (39.1)	264 (37.6)	<0.0001
>20 g/day, *n* (%)	119 (17.0)	90 (12.8)	47 (6.7)	<0.0001
Total energy intake, kJ/day	9,824.3 (8,177.4–12,170.6)	9,279.0 (7,722.2–11,155.8)	9,905.0 (8,254.4–11,951.4)	0.84
Total fat, g/day^a^	106.1 (93.8–118.5)	98.3 (88.2–108.9)	98.8 (79.7–100.2)	<0.0001
SFA, g/day^a^	40.2 (34.8–46.4)	37.0 (32.2–42.6)	32.3 (27.6–38.2)	<0.0001
SFA, en%	15.1 (12.9–17.4)	13.8 (11.7–16.3)	12.1 (10.0–14.2)	<0.0001
MUFA, g/day^a^	40.8 (35.3–48.2)	38.6 (33.6–45.7)	34.7 (29.5–41.8)	<0.0001
PUFA, g/day^a^	14.7 (11.3–19.8)	14.5 (11.3–18.8)	14.0 (10.5–18.6)	0.004
Total fibre, g/day^a^	13.4 (11.3–14.7)	18.6 (17.2–19.8)	25.2 (22.8–28.3)	<0.0001
Total fibre, g/1000 kcal	5.6 (4.9–6.2)	7.9 (7.3–8.4)	10.7 (9.8–12.2)	<0.0001
Soluble fibre, g/day^a^	4.2 (3.5–4.7)	5.8 (5.3–6.4)	7.9 (7.1–9.2)	<0.0001
Insoluble fibre, g/day^a^	8.9 (7.6–9.9)	12.5 (11.7–13.5)	17.3 (15.6–19.5)	<0.0001

Values are mean ± SD, median (IQR) or *n* (%)

^a^Nutrient intakes were adjusted for total energy intake by using the nutrient residual method [31]

### SFA and CVD risk

HR and 95% CI for fatal and non-fatal CVD according to tertiles of SFA and per 10 g/day are shown in Table 2. No statistically significant association between SFA and CVD was found, with or without adjustment for potential confounders. The association between replacement of 5% of energy from SFA by concomitant higher energy from MUFA, PUFA or CH and CVD risk was also not statistically significant (Table 3).

**Table 2:** HR for fatal and non-fatal CVD and SFA in participants in the EURODIAB PCS (*n* = 2,108)

Variable	Tertiles of SFA			*p* value for trend	Per 10 g/day SFA
	1	2	3		
Median SFA, g/day (IQR)	28.6 (24.8–31.0)	36.6 (34.9–38.5)	45.4 (42.7–49.5)		34.8 (25.7–45.5)
*n*	702	703	703		2,108
*n* cases	52	48	48		148
Person-years	5,101	5,090	5,118		15,309
Model 1^a^	1.00	1.04 (0.70, 1.57)	1.00 (0.67, 1.48)	0.99	0.95 (0.79, 1.13)
Model 2^b^	1.00	1.07 (0.70, 1.62)	1.03 (0.68, 1.55)	0.91	0.96 (0.80, 1.18)
Model 3^c^	1.00	0.95 (0.62, 1.46)	0.84 (0.53, 1.32)	0.43	0.85 (0.69, 1.05)

Values are HR (95% CI) obtained from Cox proportional hazards models according to stratification by tertiles of SFA and continuously per 10 g/day SFA. SFA was adjusted for total energy using the nutrient residual method [31]

^a^Model 1 adjusted for age (continuous), sex and energy (kJ/day)

^b^Model 2 additionally adjusted for diabetes duration (years), HbA_1c_ (%), smoking status (no, previous, current), physical activity (physical inactivity, mild physical activity ≥1 time/week, moderate physical activity ≥1 time/week, vigorous physical activity ≥1 time/week) and alcohol (0, >0–<5, 5–<15, 15–<30, 30–<40, ≥40 g/day)

^c^Model 3 additionally adjusted for total dietary fibre (g/1,000 kcal)

**Table 3:** HR for replacing 5% of energy from MUFA, PUFA or CH by 5% of energy from SFA and fatal and non-fatal CVD risk in participants in the EURODIAB PCS (*n* = 2,108, *n* cases = 148)

Model	HR	95% CI
MUFA for SFA		
Model 1^a^	1.00	0.98, 1.02
Model 2^b^	0.98	0.89, 1.07
PUFA for SFA		
Model 1^a^	1.00	0.94, 1.07
Model 2^b^	0.99	0.89, 1.09
CH for SFA		
Model 1^a^	0.98	0.94, 1.02
Model 2^b^	0.96	0.89, 1.05

Values are HR and 95% CI obtained from Cox proportional hazards models. The estimated HR for MUFA, PUFA or CH can be interpreted as replacing 5% of energy from MUFA, PUFA or CH by 5% of energy from SFA and occurrence of CVD

Nutrients were adjusted for total energy by using the nutrient residual method [31]

^a^Model 1 included age (continuous), sex, MUFA, PUFA, TFA, protein and CH (en%), and total energy (kJ/day)

^b^Model 2 additionally included diabetes duration (years), HbA_1c_ (%), smoking status (no, previous, current), physical activity (physical inactivity, mild physical activity ≥1 time/week, moderate physical activity ≥1 time/week, vigorous physical activity ≥1 time/week) and alcohol (0, >0–<5, 5–<15, 15–<30, 30–<40, ≥40 g/day)

### SFA and all-cause mortality risk

No statistically significant association between all-cause mortality and SFA was found (HR 0.70; 95% CI 0.48, 1.04) with multivariate adjustment, per 10 g/day SFA (data not shown).

### Dietary fibre and CVD risk

HR and 95% CI for fatal and non-fatal CVD are shown in Table 4. A significantly reduced CVD risk of 16% was found per 5 g/day total dietary fibre (95% CI 0.72, 0.98). For soluble fibre, a significantly decreased CVD risk of 44% (95% CI 0.35, 0.90; *p* for trend, 0.01) was found in the highest tertile, 39% (95% CI 0.38, 0.97) for each 5 g/day and 20% (95% CI 0.67, 0.97) for each 2 g/day. Additional adjustment for MUFA (g/day) and PUFA (g/day), antihypertensive use (yes or no) and daily insulin dose (units per kg) and frequency (no. of injections per day) did not alter these associations (highest tertile, HR 0.58; 95% CI 0.33, 0.93; per 5 g/day, HR 0.64; 95% CI 0.35, 0.98; per 2 g/day, HR 0.82; 95% CI 0.65, 0.98). For insoluble fibre, a significantly decreased CVD risk of 24% was found per 5 g/day (95% CI 0.61, 0.94). Adjustments for potential mediators such as systolic blood pressure, total/HDL-cholesterol ratio and BMI did not alter any of the above-mentioned associations with CVD risk and we could not identify any clear pathway (ESM Table 3).

**Table 4:** HR for fatal and non-fatal CVD according to total, soluble and insoluble fibre intake in participants in the EURODIAB PCS (*n* = 2,108)

Intake	Tertiles of fibre intake			*p* value for trend	Per 2 g/day	Per 5 g/day
	1	2	3			
*n*	702	703	703			
Total fibre						
Median intake g/day (IQR)	13.4 (11.3–14.7)	18.6 (17.2–19.8)	25.2 (22.8–28.3)			
*n* cases	50	60	38			
Person-years	5,141	5,102	5,066			
Model 1^a^	1.00	1.12 (0.77, 1.64)	0.69 (0.45, 1.06)	0.08	0.94 (0.89, 0.99)	0.86 (0.75, 0.98)
Model 2^b^	1.00	1.15 (0.81, 1.72)	0.74 (0.47, 1.14)	0.11	0.94 (0.89, 1.00)	0.87 (0.76, 0.99)
Model 3^c^	1.00	1.09 (0.78, 1.70)	0.69 (0.43, 1.11)	0.05	0.93 (0.87, 0.98)	0.84 (0.72, 0.98)
Soluble fibre						
Median intake g/day (IQR)	4.1 (3.5–4.6)	5.8 (5.4–6.3)	7.9 (7.2–9.2)			
*n* cases	55	56	37			
Person-years	5,149	5,073	5,087			
Model 1^a^	1.00	0.97 (0.67, 1.42)	0.61 (0.40, 0.93)	0.02	0.84 (0.72, 0.99)	0.66 (0.44, 0.98)
Model 2^b^	1.00	1.02 (0.69, 1.51)	0.63 (0.41, 0.98)	0.03	0.85 (0.72, 1.01)	0.69 (0.45, 1.06)
Model 3^c^	1.00	0.98 (0.66, 1.45)	0.56 (0.35, 0.90)	0.01	0.80 (0.67, 0.97)	0.61 (0.38, 0.97)
Insoluble fibre						
Median intake g/day (IQR)	8.9 (7.6–9.9)	12.5 (11.7–13.5)	17.3 (15.6–19.5)			
*n* cases	48	57	43			
Person-years	5,144	5,103	5,062			
Model 1^a^	1.00	1.08 (0.73, 1.60)	0.80 (0.53, 1.22)	0.26	0.92 (0.85, 0.99)	0.80 (0.66, 0.97)
Model 2^b^	1.00	1.09 (0.74, 1.62)	0.83 (0.54, 1.27)	0.34	0.92 (0.85, 0.99)	0.81 (0.67, 0.99)
Model 3^c^	1.00	1.05 (0.71, 1.57)	0.76 (0.48, 1.21)	0.21	0.89 (0.81, 0.97)	0.76 (0.61, 0.94)

Values are HR (95% CI) obtained from Cox proportional hazards models according to stratification by tertiles of total, soluble and insoluble fibre intake and continuously per 2 g/day and 5 g/day increase in total, soluble and insoluble fibre intake. Fibre intake was adjusted for total energy intake by using the nutrient residual method [31]

^a^Model 1 adjusted for age (continuous), sex and energy intake (kJ/day)

^b^Model 2 additionally adjusted for diabetes duration (years), HbA_1c_ (%), smoking status (no, previous, current), physical activity (physical inactivity, mild physical activity ≥1 time/week, moderate physical activity ≥1 time/week, vigorous physical activity ≥1 time/week) and alcohol intake (0, >0–<5, 5–<15, 15–<30, 30–<40, ≥40 g/day)

^c^Model 3 additionally adjusted for SFA intake (en%)

### Dietary fibre and all-cause mortality risk

A significant inverse association was found after adjustment for potential confounders including age, sex, energy intake, diabetes duration, HbA_1c_, smoking status, physical activity, alcohol and SFA for the relationship between dietary fibre and all-cause mortality. A significantly decreased all-cause mortality risk of 28% was found for each 5 g/day total dietary fibre (95% CI 0.55, 0.95; Table 5). The association with all-cause mortality was stronger for soluble fibre (HR 0.34; 95% CI 0.14, 0.80) than for insoluble fibre (HR 0.66; 95% CI 0.45, 0.97; Table 5). These associations became stronger after additional adjustments for MUFA (g/day) and PUFA (g/day), antihypertensive use (yes or no), and daily insulin dose (units/kg) and frequency (no. of injections per day; soluble fibre, HR 0.28; 95% CI 0.11, 0.68; insoluble fibre, HR 0.60; 95% CI 0.40, 0.90). A significantly decreased all-cause mortality risk of 37% was found per 2 g/day soluble fibre (95% CI 0.45, 0.87). Adjustments for potential mediators such as systolic blood pressure, total/HDL-cholesterol ratio and BMI did not alter any of the above-mentioned associations with all-cause mortality risk (ESM Table 4).

**Table 5:** HR for all-cause mortality according to each increase in 2 g/day and 5 g/day total, soluble and insoluble fibre intake in participants in the EURODIAB PCS (*n* = 2,108, *n* cases = 46)

Intake	Per 2 g/day	Per 5 g/day
Total fibre		
Model 1^a^	0.93 (0.85, 1.02)	0.84 (0.67, 1.06)
Model 2^b^	0.92 (0.83, 1.01)	0.83 (0.65, 1.05)
Model 3^c^	0.87 (0.78, 0.97)	0.72 (0.55, 0.95)
Soluble fibre		
Model 1^a^	0.78 (0.58, 1.04)	0.53 (0.26, 1.11)
Model 2^b^	0.73 (0.54, 1.00)	0.51 (0.24, 1.10)
Model 3^c^	0.63 (0.45, 0.87)	0.34 (0.14, 0.80)
Insoluble fibre		
Model 1^a^	0.92 (0.81, 1.05)	0.81 (0.58, 1.12)
Model 2^b^	0.90 (0.79, 1.03)	0.79 (0.57, 1.11)
Model 3^c^	0.84 (0.72, 0.97)	0.66 (0.45, 0.97)

Values are HR and 95% CI obtained from Cox proportional hazards models according to each 2 g/day and 5 g/day increase in total, soluble and insoluble fibre. Fibre intake was adjusted for total energy intake (kJ/day) using the nutrient residual method [31]

^a^Model 1 adjusted for age (continuous), sex and energy (kJ/day)

^b^Model 2 additionally adjusted for diabetes duration (years), HbA_1c_ (%), smoking status (no, previous, current), physical activity (physical inactivity, mild physical activity ≥1 time/week, moderate physical activity ≥1 time/week, vigorous physical activity ≥1 time/week) and alcohol intake (0, >0–<5, 5–<15, 15–<30, 30–<40, ≥40 g/day)

^c^Model 3 additionally adjusted for SFA intake (en%)

## Discussion

The results of the present study among 2,108 European patients with type 1 diabetes suggest an inverse association between dietary fibre and CVD risk and all-cause mortality, with a stronger protective association for soluble fibre compared with insoluble fibre. No statistically significant association was found between all-cause mortality risk and SFA and CVD, nor between all-cause mortality risk and replacing 5% of energy from SFA by concomitant higher energy from PUFA, MUFA or CH.

The present findings on SFA support the results of previous studies in the general population [15, 16] demonstrating that there is no statistically significant relationship between SFA and CVD risk. However, a cross-sectional study performed among children and adolescents with type 1 diabetes showed that a diet high in energy from SFA was associated with a higher risk of CVD [11]. A recent meta-analysis of randomised controlled trials [33] and a pooled analysis of 11 cohort studies [34] both found consistent evidence in the general population of decreased CVD risk when SFA was replaced by PUFA. In contrast with these studies, we did not find any association with CVD risk when SFA was replaced by MUFA, PUFA or CH, which might be explained by a lack of power in our study.

Our results on dietary fibre confirm the results of a cross-sectional study among type 1 diabetic patients which showed that a diet low in fibre, fruit and vegetables could lead to a higher risk of CVD [11]. Also, results from cohort studies in the general population suggesting an inverse association between dietary fibre and CVD risk are in line with our results [19, 20]. We found a significantly lower CVD risk of 32% per 10 g/day total dietary fibre (95% CI 0.48, 0.95), which is in accordance with the pooled analyses of cohort studies in the general population by Pereira et al. [19]. Such a change in total dietary fibre is challenging to achieve; however, a more realistic change in dietary fibre of 5 g/day was significantly associated with a 16% lower risk in our study (95% CI 0.72, 0.98). Some studies on whether protection of dietary fibre may come from soluble and/or insoluble fibre show no clear advantage of either type of dietary fibre [35, 36], whereas the pooled analysis of Pereira et al. shows a stronger inverse association for soluble fibre [19]. These authors showed a lower coronary heart disease risk of 44% per 10 g/day, whereas we found in type 1 diabetic patients a lower CVD risk of 67% per 10 g/day soluble fibre (95% CI 0.13, 0.88). Again, 10 g/day soluble fibre may be unfeasible; however, changing this to 2 g/day soluble fibre showed a statistically significantly lower CVD risk of 18% in our study (95% CI 0.67, 0.97). Besides the inverse association between dietary fibre and CVD risk, we observed a significant inverse association with all-cause mortality, which is in line with results found in the general population [37], as well as in patients with type 2 diabetes [21]. In the Nurses’ Health Study [21], bran intake in the highest quintile was associated with a 28% (95% CI 0.56, 0.92) lower all-cause mortality risk and a 35% (95% CI 0.43, 0.99) lower CVD-specific mortality risk in type 2 diabetic patients. In the Zutphen Study [37], no clear difference between the effects of dietary fibre from various sources was observed. Park et al. [38] showed in a non-diabetic population a significant inverse association with all-cause mortality from total dietary fibre and grains, but not from other sources, whereas we showed significant inverse associations in type 1 diabetic patients for total, soluble and insoluble fibre. Most likely, as assessed in prospective studies, dietary fibre within the usual diet is a marker of healthy food choices with an overall cardiovascular benefit [19]. However, in our study, we were able to demonstrate an independent protective association between dietary fibre and CVD risk in type 1 diabetic patients, after adjustment for lifestyle factors characterising a healthy lifestyle.

The present study is, to our knowledge, the first large prospective cohort study with a sufficient number of CVD cases and deaths in young type 1 diabetic patients to report the associations between SFA and dietary fibre and risk of CVD and all-cause mortality in this population. The observed relationships were independent of sex, age, energy intake, CVD risk factors and other dietary factors. The strength of the study lies in its large sample size of European type 1 diabetic patients. Furthermore, the EURODIAB PCS provides a multicentre, clinic-based, prospective study, with the same standardised methods used in each centre. However, some limitations should be addressed. First, the accuracy of dietary data and the absence of data on foods are important limitations. The use of information on dietary intake from self-reported 3 day dietary records may be subject to the weakness of the nutritional data [26]. Measurement error is always an inherent limitation in self-reported dietary assessment and has an impact on results, conclusions and interpretation. Under the assumption that measurement error is non-differential with regard to the outcome, this might have led to biased risk estimates and reduced statistical power for detecting associations. Second, 1,142 participants were excluded from the present study due to loss to follow-up and missing information on dietary intake, potential confounders, and CVD and/or all-cause mortality at follow-up. Loss to follow-up due to no participation at follow-up and no information on CVD and/or all-cause mortality at follow-up might have caused a selection bias which could have modified the associations under study. We compared those included in the study with those lost to follow-up and found that patients with a more atherosclerotic risk profile were lost, which may imply that our demonstrated associations for dietary fibre and CVD or all-cause mortality could have been even stronger if these patients had been included. Furthermore, although we minimised the influence of confounding in the associations by adjustment for key confounders, residual confounding cannot be ruled out due to interference by factors we did not measure. Finally, upon diagnosis many patients might have received dietary advice from dietitians and physicians at diabetes clinics which might have introduced bias in our results. However, this study gives insight into the real-life course of the disease.

Possible reasons for not finding any association between SFA and blood cholesterol and CVD could be inaccurate dietary methods; large day-to-day variations; chain length of SFA, animal or plant source; natural or interesterified forms of SFA [39]. Replacement of SFA by PUFA has indeed been shown to be effective in reducing CVD risk in both meta-analyses of prospective studies [34] and in controlled feeding trials [33]; therefore recommendations should remain unaltered with the advice to restrict SFA intake below 7% of total energy intake, particularly in patients at increased risk of CVD.

Several mechanisms have been suggested by which dietary fibre may reduce CVD risk in the general population. Several studies have found that increased dietary fibre may lower the risk of type 2 diabetes [40–42], which may partly explain our associations with CVD and all-cause mortality [21]. Including potential mediators such as systolic blood pressure, serum cholesterol and BMI did not lead to identification of a clear pathway. Soluble fibre might be able to delay the absorption of nutrients and bind bile acids in the small intestine, which may increase bile acid excretion [43]. These effects have been shown to lower total and LDL-cholesterol levels [44] and improve insulin sensitivity [45]. This in turn is associated with reductions in blood pressure [46]. In addition, soluble fibre-containing foods such as fruit and vegetables have been shown to slow down or reduce glucose absorption in the intestine due to a reduction in the glycaemic index [47, 48]. Results from the EURODIAB PCS show that total dietary fibre was significantly inversely associated with HbA_1c_ levels, independently of other lifestyle and nutritional factors [49].

In conclusion, our results in European individuals with type 1 diabetes do not show a statistically significant association between SFA and CVD risk. By contrast, higher total, soluble and insoluble dietary fibre appears to be strongly protective of CVD and all-cause mortality in our population. These results are in line with the current dietary guidelines for diabetic patients on dietary fibre-rich foods [9, 50]: to encourage dietary fibre intake from wholegrains, fruit, vegetables and various sources that are easily available and not too costly in the different European countries. Trials of dietary intervention in type 1 diabetes should focus on acceptable methods to increase dietary fibre intake, and determine the effects on CVD and all-cause mortality risk. Moreover, considering limitations of dietary assessment methods, we recommend further research by other studies and designs investigating the role of dietary SFA and fibre and the risk of CVD and all-cause mortality in type 1 diabetic patients.

## Electronic supplementary material

Below is the link to the electronic supplementary material.

ESM Table 1 (PDF 78.7 kb)

ESM Table 2 (PDF 94.2 kb)

ESM Table 3 (PDF 106 kb)

ESM Table 4 (PDF 79 kb)

ESM List of members of the EURODIAB Prospective Complications Study Group (PDF 148 kb)

## Abbreviations

CH: Carbohydrates
CVD: Cardiovascular disease
en%: Energy percentage
IQR: Interquartile range
MUFA: Monounsaturated fatty acids
PCS: Prospective Complications Study
PUFA: Polyunsaturated fatty acids
SFA: Saturated fatty acids
TFA: *trans*-Fatty acids
