# Supplementary material for: Dietary saturated fat and fibre and risk of cardiovascular disease and all-cause mortality among type 1 diabetic patients: the EURODIAB Prospective Complications Study
Source: Diabetologia. 2012 Apr 12;55(8):2132–41. doi: 10.1007/s00125-012-2550-0 (PMC3390695; doi:10.1007/s00125-012-2550-0)
Supplement: Supplementary file 1 — (PDF 78.7 kb) [file 125_2012_2550_MOESM1_ESM.pdf]

Electronic supplementary material

ESM Table 1 Baseline CVD risk factors for participants in the EURODIAB PCS included and excluded for the present study

	Study participants	Excluded participants	*p*-value[a]
n	2,108	1,142	
Age, years	32.1 ± 9.7	33.8 ± 9.8	<0.0001
Sex, *n* (%) male	1,081 (51.3)	570 (49.9)	0.97
Diabetes duration, years	14.3 ± 9.0	15.4 ± 9.2	0.003
Daily insulin dose, units/kg	0.7 ± 0.2	0.7 ± 0.2	0.55
Daily insulin frequency, injections per day	2.6 ± 0.7	2.7 ± 0.7	0.07
Hba_{1c}, % (mmol/mol)	8.3 ± 1.9 (67.2)	8.6 ± 2.0 (70.5)	0.01
Systolic blood pressure, mmHg	120.0 ± 16.8	123.7 ± 17.3	<0.0001
Diastolic blood pressure, mmHg	75.1 ± 11.2	76.2 ± 11.3	0.08
Antihypertensive use, *n* (%)	160 (7.6)	171 (15.0)	<0.0001
BMI, kg/m^2	23.5 ± 2.8	23.8 ± 3.2	<0.0001
Triacylglycerol, mmol/l	1.1 ± 0.7	1.3 ± 0.7	<0.0001
Cholesterol, mmol/l	5.3 ± 1.1	5.4 ± 1.2	0.05
HDL-cholesterol, mmol/l	1.5 ± 0.4	1.4 ± 0.6	0.03
LDL-cholesterol, mmol/l	3.3 ± 1.0	3.4 ± 1.0	0.26
Total:HDL-cholesterol ratio, mmol/l	3.5 ± 1.2	3.9 ± 1.6	<0.0001
Moderate physical activity ≥1 time/week, *n* (%)	1,427 (67.7)	726 (63.6)	0.02
Ever smoker, *n* (%)	1,018 (48.3)	605 (53.0)	0.03
Total energy intake, kJ/day	10,063.5 ± 2,911.5	9,589.6 ± 3,102.3	0.03
Alcohol intake, g/day	7.6 ± 15.6	5.9 ± 15.7	<0.0001
Total fat, g/day[b]	97.9 ± 36.0	100.5 ± 36.6	<0.0001
Totat fat, en%	37.5 ± 7.1	39.0 ± 8.0	0.02
SFA, g/day[b]	36.9 ± 15.6	38.5 ± 16.5	0.0002
SFA, en%	13.7 ± 3.4	14.6 ± 3.6	0.00
PUFA, g/day[b]	15.9 ± 9.5	16.9 ± 10.3	0.004
MUFA, g/day[b]	36.4 ± 15.2	39.4 ± 15.3	<0.0001
Total fibre, g/day[b]	19.2 ± 7.5	17.1 ± 6.9	<0.0001
Total fibre, g/1000 kcal	8.7 ± 3.9	7.6 ± 3.8	<0.0001
Soluble fibre, g/day[b]	6.0 ± 2.4	5.6 ± 1.3	<0.0001
Insoluble fibre, g/day[b]	13.1 ± 5.2	11.5 ± 4.9	<0.0001

[a] *p* values from Chi-squared test

[b] Nutrient intakes were adjusted for total energy intake by using the nutrient residual method (31).
