# Supplementary material for: Dietary saturated fat and fibre and risk of cardiovascular disease and all-cause mortality among type 1 diabetic patients: the EURODIAB Prospective Complications Study
Source: Diabetologia. 2012 Apr 12;55(8):2132–41. doi: 10.1007/s00125-012-2550-0 (PMC3390695; doi:10.1007/s00125-012-2550-0)
Supplement: Supplementary file 2 — (PDF 94.2 kb) [file 125_2012_2550_MOESM2_ESM.pdf]

Electronic supplementary material

ESM Table 2 Baseline characteristics of type 1 diabetic patients in the EURODIAB PCS according to tertiles of SFA intake (*n*=2,108)

Characteristic	Tertiles of SFA intake[a]			*p*-value for trend
	1	2	3	
n	702	703	703	
Mean follow-up, years	7.3 ± 1.1	7.2 ± 1.1	7.3 ± 0.9	0.81
Age, years	32.7 ± 9.8	31.7 ± 9.9	31.9 ± 9.5	0.13
Sex, *n* (%) male	438 (62.4)	314 (44.7)	329 (46.8)	<0.0001
Diabetes duration, years	12.6 (6.6-19.4)	12.9 (7.4-18.6)	13.6 (8.0-19.9)	<0.0001
HbA_{1c}, % (mmol/mol)	8.0 ± 2.0 (63.9)	8.1 ± 2.0 (65.0)	8.1 ± 1.8 (65.0)	0.44
Daily insulin dose, units/kg	0.7 (0.5-0.8)	0.7 (0.5-0.8)	0.7 (0.6-0.8)	0.92
Daily insulin frequency, injections/day	3.0 (2.0-3.0)	2.0 (2.0-3.0)	2.0 (2.0-3.0)	0.02
Systolic blood pressure, mmHg	120.8 ± 17.0	119.3 ± 16.9	119.9 ± 16.3	0.31
Diastolic blood pressure, mmHg	74.7 ± 11.6	75.2 ± 11.3	75.3 ± 10.7	0.27
Antihypertensive use, *n* (%)	57 (8.2)	58 (8.3)	44 (6.3)	0.17
BMI, kg/m^2	23.4 ± 2.7	23.6 ± 2.8	23.5 ± 2.8	0.38
Triacylglycerol, mmol/l	0.9 (0.7-1.2)	0.9 (0.7-1.3)	0.9 (0.7-1.2)	0.57
Total cholesterol, mmol/l	5.3 ± 1.2	5.3 ± 1.1	5.3 ± 1.1	0.27
HDL-cholesterol, mmol/l	1.5 ± 0.4	1.5 ± 0.4	1.5 ± 0.4	0.77
LDL-cholesterol, mmol/l	3.3 ± 1.0	3.4 ± 1.0	3.3 ± 1.0	0.92
Total:HDL-cholesterol ratio, mmol/l	3.4 ± 1.4	3.4 ± 1.1	3.3 ± 1.2	0.15
Physical inactivity, *n* (%)	27 (3.9)	17 (3.4)	16 (2.3)	<0.0001
Mild physical activity ≥1 time/week, *n* (%)	222 (32.2)	234 (33.6)	210 (30.3)	<0.0001
Moderate physical activity ≥1 time/week, *n* (%)	202 (29.3)	237 (34.1)	253 (36.5)	<0.0001
Vigorous physical activity ≥1 time/week, *n* (%)	239 (34.6)	208 (29.9)	214 (30.9)	<0.0001
Ever smoker, *n* (%)	342 (48.7)	326 (46.4)	350 (49.8)	0.11
Alcohol intake, g/day	2.5 (0.0-16.1)	0.0 (0.0-7.3)	0.0 (0.0-7.0)	<0.0001
None, *n* (%)	289 (41.2)	374 (53.2)	360 (51.2)	<0.0001
≤ 20 g/day, *n* (%)	281 (40.0)	261 (37.1)	287 (40.8)	0.19
>20 g/day, *n* (%)	132 (18.8)	68 (9.7)	56 (8.0)	<0.0001
Total energy intake, kJ/day	10,247.0 (8,513.9-12,327.4)	8,814.4 (7,360.0-10,989.2)	9,934.1 (8,331.8-12,139.4)	0.09
Total fat, g/day[a]	83.9 (73.0-92.7)	96.8 (89.0-104.8)	110.5 (102.5-121.1)	<0.0001
SFA, g/day[a]	28.6 (24.8-31.0)	36.6 (34.938.5)	45.4 (42.7-49.5)	<0.0001
SFA, en%	10.6 (9.3-11.5)	13.6 (12.9-14.4)	17.2 (16.2-18.3)	<0.0001
MUFA, g/day[a]	35.1 (28.8-42.4)	37.2 (32.2-44.4)	41.2 (36.2-47.9)	<0.0001
PUFA, g/day[a]	12.2 (9.317.8)	15.1 (12.0-19.4)	15.3 (12.2-19.6)	<0.0001
Total fibre, g/day[a]	21.8 (17.1-26.2)	18.8 (15.1-22.0)	16.1 (12.9-19.3)	<0.0001
Total fibre, g/1000kcal	9.2 (7.4-11.0)	8.0 (6.3-9.6)	6.8 (5.5-8.2)	<0.0001
Soluble fibre, g/day[a]	6.8 (5.4-8.3)	5.8 (4.7-7.1)	5.1 (4.0-6.2)	<0.0001
Insoluble fibre, g/day[a]	14.9 (11.8-18.1)	12.6 (10.2-15.1)	10.8 (8.8-13.2)	<0.0001

Values are mean ± SD, median (IQR) or *n* (%)

[a] Nutrient intakes were adjusted for total energy intake by using the nutrient residual method (31).
