# Supplementary material for: Dietary saturated fat and fibre and risk of cardiovascular disease and all-cause mortality among type 1 diabetic patients: the EURODIAB Prospective Complications Study
Source: Diabetologia. 2012 Apr 12;55(8):2132–41. doi: 10.1007/s00125-012-2550-0 (PMC3390695; doi:10.1007/s00125-012-2550-0)
Supplement: Supplementary file 3 — (PDF 106 kb) [file 125_2012_2550_MOESM3_ESM.pdf]

Electronic supplementary material

ESM Table 3 HR for fatal and nonfatal CVD according to total, soluble, and insoluble fibre intake in participants in the EURODIAB PCS: additional adjustments for potential mediators (*n*=2,108)

Intake		Tertiles of fibre intake			*p*-value		
		1	2	3	for trend	Per 2 g/day	Per 5 g/day
	n	702	703	703			
Total fibre	Median intake g/day (IQR)	13.4 (11.3-14.7)	18.6 (17.2-19.8)	25.2 (22.8-28.3)			
	n cases	50	60	38			
	Person-years	5,141	5,102	5,066			
	Model 1[a]	1.00	1.09 (0.78, 1.70)	0.69 (0.43, 1.11)	0.05	0.93 (0.72, 0.98)	0.84 (0.72, 0.98)
	Model 2[b]	1.00	1.03 (0.65, 1.63)	0.69 (0.40, 1.21)	0.05	0.91 (0.84, 0.98)	0.80 (0.66, 0.97)
	Model 3[c]	1.00	1.12 (0.76, 1.66)	0.67 (0.42, 1.08)	0.03	0.93 (0.87, 0.99)	0.84 (0.72, 0.99)
	Model 4[d]	1.00	1.16 (0.79, 0.72)	0.69 (0.43, 1.11)	0.03	0.93 (0.87, 0.99)	0.85 (0.73, 0.99)
Soluble fibre	Median intake g/day (IQR)	4.1 (3.5-4.6)	5.8 (5.4-6.3)	7.9 (7.2-9.2)			
	n cases	55	56	37			
	Person-years	5,149	5,073	5,087			
	Model 1[a]	1.00	0.98 (0.66, 1.45)	0.56 (0.35, 0.90)	0.01	0.80 (0.67, 0.97)	0.61 (0.38, 0.97)
	Model 2[b]	1.00	1.01 (0.64, 1.61)	0.56 (0.32, 0.98)	0.01	0.79 (0.63, 0.99)	0.55 (0.31, 0.98)
	Model 3[c]	1.00	0.97 (0.65, 1.44)	0.55 (0.34, 0.88)	0.01	0.82 (0.68, 0.99)	0.61 (0.38, 0.98)
	Model 4[d]	1.00	0.99 (0.67, 1.46)	0.57 (0.35, 0.91)	0.01	0.83 (0.68, 0.99)	0.62 (0.39, 0.99)
Insoluble fibre	Median intake g/day (IQR)	8.9 (7.6-9.9)	12.5 (11.7-13.5)	17.3 (15.6-19.5)			
	n cases	48	57	43			
	Person-years	5,144	5,103	5,062			
	Model 1[a]	1.00	1.05 (0.71, 1.57)	0.76 (0.48, 1.21)	0.21	0.89 (0.81, 0.97)	0.76 (0.61, 0.94)
	Model 2[b]	1.00	1.01 (0.64, 1.62)	0.75 (0.43, 1.31)	0.30	0.87 (0.78, 0.98)	0.71 (0.54, 0.94)
	Model 3[c]	1.00	1.05 (0.71, 1.57)	0.73 (0.45, 1.16)	0.19	0.89 (0.82, 0.97)	0.75 (0.60, 0.94)
	Model 4[d]	1.00	1.06 (0.71, 1.58)	0.77 (0.48, 1.22)	0.20	0.90 (0.82, 0.98)	0.76 (0.61, 0.95)

Values are HR (95% CIs) obtained from Cox proportional hazards models according to stratification by tertiles of total, soluble, and insoluble fibre intake and continuously per 2 g/day and 5 g/day increase in total, soluble, and insoluble fibre intake. Fibre intake was adjusted for total energy intake (kJ/day) by using the nutrient residual method (31).

[a] Model 1 adjusted for age (continuous), sex and energy intake (kJ/day), diabetes duration (years), HbA_{1c} (%), smoking status (no, previous, current), physical activity (physical inactivity, mild PA ≥1 time/week, moderate PA ≥1 time/week and vigorous PA ≥1 time/week), alcohol intake (0, >0-<5, 5-<15, 15-<30, 30-<40, and ≥40 g/day), and SFA intake (en%)

[b] Model 2 additionally adjusted for Total:HDL-cholesterol ratio (mmol/l)

[c] Model 3 additionally adjusted for systolic and diastolic blood pressure (mmHg)

[d] Model 4 additionally adjusted for BMI (BMI <25 kg/m^2, BMI 25-30 kg/m^2, and BMI >30 kg/m^2)
