# Supplementary material for: Dietary saturated fat and fibre and risk of cardiovascular disease and all-cause mortality among type 1 diabetic patients: the EURODIAB Prospective Complications Study
Source: Diabetologia. 2012 Apr 12;55(8):2132–41. doi: 10.1007/s00125-012-2550-0 (PMC3390695; doi:10.1007/s00125-012-2550-0)
Supplement: Supplementary file 4 — (PDF 79 kb) [file 125_2012_2550_MOESM4_ESM.pdf]

Electronic supplementary material

ESM Table 4 HR for all-cause mortality according to each increase of 2 g/day and 5 g/day total, soluble, and insoluble fibre intake in participants in the EURODIAB PCS: additional adjustments for potential mediators (*n*=2,108, *n* cases=46)

Intake		Per 2 g/day	Per 5 g/day
Total fibre	Model 1[a]	0.87 (0.78, 0.95)	0.72 (0,55, 0,95)
	Model 2[b]	0.80 (0.68, 0.94)	0,57 (0.39, 0.85)
	Model 3[c]	0.88 (0,78, 0,98)	0.72 (0.54, 0.95)
	Model 4[d]	0.88 (0.79, 0.98)	0.72 (0.55, 0.95)
Soluble fibre	Model 1[a]	0.63 (0.45, 0,87)	0.34 (0.14, 0.80)
	Model 2[b]	0.50 (0.31, 0.80)	0.17 (0.05, 0.58)
	Model 3[c]	0.64 (0.46, 0.91)	0.33 (0.14, 0.79)
	Model 4[d]	0.65 (0.46, 0.91)	0.34 (0.14, 0.80)
Insoluble fibre	Model 1[a]	0.84 (0.72, 0.97)	0.66 (0.45, 0.97)
	Model 2[b]	0.76 (0.61, 0.94)	0.50 (0.29, 0.87)
	Model 3[c]	0.85 (0.73, 0.99)	0.66 (0.45, 0.97)
	Model 4[d]	0.85 (0.73, 0.99)	0.66 (0.45, 0.97)

Values are HRs and 95% CIs obtained from Cox proportional hazards models according to each 2 g/day and 5 g/day increase in total, soluble, and insoluble fibre. Fibre intake was adjusted for total energy intake (kJ/day) using the nutrient residual method (31).

[a] Model 1 adjusted for age (continuous), sex and energy intake (kJ/day), diabetes duration (years), HbA_{1c} (%), smoking status (no, previous, current), physical activity (physical inactivity, mild PA ≥1 time/week, moderate PA ≥1 time/week and vigorous PA ≥1 time/week), alcohol intake (0, >0-<5, 5-<15, 15-<30, 30-<40, and ≥40 g/day), and SFA intake (en%)

[b] Model 2 additionally adjusted for Total:HDL-cholesterol ratio (mmol/l)

[c] Model 3 additionally adjusted for systolic and diastolic blood pressure (mmHg)

[d] Model 4 additionally adjusted for BMI (BMI <25 kg/m^2, BMI 25-30 kg/m^2, and BMI >30 kg/m^2)
