# Supplementary material for: Dietary saturated fat and fibre and risk of cardiovascular disease and all-cause mortality among type 1 diabetic patients: the EURODIAB Prospective Complications Study
Source: Diabetologia. 2012 Apr 12;55(8):2132–41. doi: 10.1007/s00125-012-2550-0 (PMC3390695; doi:10.1007/s00125-012-2550-0)
Supplement: Supplementary file 5 — List of members of the EURODIAB Prospective Complications Study Group (PDF 148 kb) [file 125_2012_2550_MOESM5_ESM.pdf]

Electronic supplementary material

List of members of the EURODIAB Prospective Complications Study Group

B. Karamanos, A. Kofinis, K. Petrou (Hippokration Hospital, Athens, Greece); F. Giorgino, G. Picca, A. Angarano, G. De Pergola, L. Laviola, R. Giorgino (Internal Medicine, Endocrinology and Metabolic Diseases, Department of Emergency and Organ Transplantation, University of Bari, Bari, Italy); C. Ionescu-Tirgoviste, A. Coszma, C. Guja (Clinic of Diabetes, Nutrition and Metabolic Diseases, Bucharest, Romania); M. Songini, A. Casu, M. Pedron, S. Pintus, M. Fossarello (Diabetes Unit Ospedale San Michele, Cagliari, Italy); J. B. Ferriss, G. Grealy, D. O'Keefe (Cork University Hospital, Cork, Ireland); M. Toeller, C. Arden (Diabetes Research Institute, Heinrich-Heine University, Duesseldorf, Germany); R. Rottiers, C. Tuyttens, H. Priem (University Hospital of Gent, Belgium); P. Ebeling, M. Kylliäinen , V. A. Koivisto (University Hospital of Helsinki, Finland); B. Idzior-Walus, J. Sieradzki, K. Cyganek, B. Solnica (Department of Metabolic Diseases, Jagiellonian University, Krakow, Poland); H. H. P. J. Lemkes, J. C. Lemkes-Stuffken (Leiden University Medical Centre, the Netherlands); J. Nunes-Correa, M. C. Rogado, L. Gardete-Correia, M. C. Cardoso, A. Silva, J. Boavida, M. Machado Sa Marques (Portuguese Diabetic Association, Lisbon, Portugal); G. Michel, R. Wirion, S. Cardillo (Centre Hospitalier, Luxembourg); G. Pozza, R. Mangili, V. Asnaghi (Ospedale San Raffaele, Milan, Italy); E. Standl, B. Schaffler, H. Brand, A. Harms (City Hospital Schwabing, Munich, Germany); D. Ben Soussan, O. Verier-Mine, P. Fallas, M. C. Fallas (Centre Hospitalier de Valenciennes, France); J. H. Fuller, J. Holloway, L. Asbury, D. J. Betteridge (University College London, UK); G. Cathelineau, A. Bouallouche, B. Villatte Cathelineau (Hospital Saint-Louis, Paris, France); F. Santeusanio, G. Rosi, V. D'Alessandro, C. Cagini, P. Bottini, G. P. Reboldi (Dipartimento di Medicina Interna, Perugia, Italy); R. Navalesi, G. Penno, S. Bandinelli, R. Miccoli, M. Nannipieri (Dipartimento di Endocrinologia e Metabolismo, Pisa, Italy); G. Ghirlanda, C. Saponara, P. Cotroneo, A. Manto, A. Minnella (Universita Cattolica del Sacro Cuore, Rome, Italy); J. D. Ward, S. Tesfaye, S. Eaton, C. Mody (Royal Hallamshire Hospital, Sheffield, UK); M. Borra, P. Cavallo Perin, S. Giunti, G. Grassi, G. F. Pagano, M. Porta, R. Sivieri, F. Vitelli, M. Veglio (Dipartimento di Medicina Interna, Università di Torino and ASO CTO/CRF/Maria Adelaide, Turin, Italy); N. Papazoglou, G. Manes (General Hospital Papageorgiou, Diabetes Unit, Thessaloniki, Greece); M. Muggeo, M. Iagulli, V. Cacciatori (V. Cattedra di Malattie del Metabolismo, Verona, Italy); K. Irsigler, H. Abrahamian (Hospital Vienna Lainz, Austria); S. Walford, J. Sinclair, S. Hughes, V. McLelland, J. Ward (New Cross Hospital, Wolverhampton, UK); G. Roglic, Z. Metelko, Z. R. Pepeonik (Vuk Vrhovac Institute for Diabetes, Zagreb, Croatia);

Steering Committee members

J. H. Fuller (London),B. Karamanos, Chairman (Athens), A.-K. Sjolie (Odense), N. Chaturvedi (London), M. Toeller (Duesseldorf), G. Pozza Co-chairman (Milan), B. Ferriss (Cork), M. Porta (Turin), R. Rottiers (Gent), G. Michel (Luxembourg)

Co-ordinating Centre
J. H. Fuller, N. Chaturvedi, J. Holloway, D. Webb, L. Asbury, University College London, UK

Central laboratories
G.-C. Viberti, R. Swaminathan, P. Lumb, A. Collins, S. Sankaralingham, MA Crook, Guy's and St Thomas Hospital, London, UK

Retinopathy Grading Centre
S. Aldington, T. Mortemore, H. Lipinski, Royal Postgraduate Medical School of Imperial College London, London, UK

Nutrition Co-ordinating Centre
M. Toeller, W.A. Scherbaum, F.A. Gries, Heinrich-Heine-University, Diabetes Research Institute and Department of Endocrinology, Diabetology and Rheumatology, Duesseldorf, Germany
